# 

**Published:** 1890-12

**Authors:** 


### CONTENTS.

ORIGINAL COMMUNICATIONS—

The Motive and Method of Pelvic Surgery. By Joseph Price, M. D., Philadelphia.......... 579

Case of Remarkable Injury with Recovery. By E. A. Cobleigh, M. D.......... 587

Case Report—Extra-Uterine, Pregnancy, Four Years and Eight Months' Duration, Complicated by Entero-Uterine Fistula. By R. R. Kime, M. D.......... 598

Was It Relapsing Fever? By A. D. Barr, M. D.......... 602

Abstract of Dr. R. B. Maury's Paper.......... 605

A Plea for the More Extended Use of Spectacles as a Therapeutic Measure.......... 606

Report of a Case of Hemorrhagic Malarial Fever. By J. R. G. Howell.......... 609

CORRESPONDENCE—

Our New York Letter.......... 611

SOCIETY REPORTS—

Tri-State Medical Association.......... 616

BOOK REVIEWS—

Familiar Forms of Nervous Diseases.......... 634

A Text-Book of Comparative Physiology.......... 635

EDITORIAL—

Koch's New Treatment for Tubercolosis.......... 636

A "Non-Poisonous, Non-Irritating Dressing".......... 637

The Third Annual Meeting of the Southern Surgical and Gynecological Association.......... 639

The Formal Opening of the New Academy of Medicine.......... 640

Items..........640–642

Index to Advertisements.......... xix

Reading Notices..........xviii

Premium List..........xxxiv-xxxv

